# Supplementary material for: Improved Antibody Detection for Canine Leptospirosis: ELISAs Modified Using Local Leptospiral Serovar Isolates from Asymptomatic Dogs
Source: Animals (Basel). 2024 Mar 14;14(6):893. doi: 10.3390/ani14060893 (PMC10967358; doi:10.3390/ani14060893)
Supplement: Supplementary file 1 [file animals-14-00893-s001.zip › animals-2887130-supplementary.pdf]

## Supplementary Materials

**Supplementary Table S1.** The number and percentage of 260 sera from five dog groups that tested positive by modified ELISAs (antibody detection) using other four local isolates of leptospiral serovars and four common *Leptospira* serovars used in the leptospirosis vaccine for dogs.

| Groups | Group 1 | Group 2 | Group 3 | | | Group 4 | Group 5 | Total |
|---|---|---|---|---|---|---|---|---|
| | Infected dogs from Nan province confirmed by positive PCR and isolation | Unvaccinated dogs from Nan province | Vaccinated dogs from Bangkok | | | Unvaccinated dogs from non-endemic areas | Unvaccinated puppies from non-endemic areas | |
| **Number of samples** | 6 | 21 | 23* | 89** | 112 | 108 | 13 | 260 |
| **Methods (modified ELISAs), protein preparation, and local isolates of serovars used in the ELISAs** | | | | | | | | |
| **WCP-Paidjan/IgG-ELISA** | 6 (6/6; 100%) | 13 (13/21; 62%) | 5 (5/23; 22%) | 18 (18/89; 20%) | 23 (23/112; 21%) | 0 (0/108; 0%) | 0 (0/13; 0%) | **42 (42/260; 16%)** |
| **WCP-Bataviae/IgG-ELISA** | 6 (6/6; 100%) | 14 (14/21; 67%) | 1 (1/23; 4%) | 5 (5/89; 6%) | 6 (6/112; 5%) | 0 (0/108; 0%) | 0 (0/13; 0%) | **26 (26/260; 10%)** |
| **WCP-Mini06/IgG-ELISA** | 6 (6/6; 100%) | 13 (13/21; 62%) | 1 (1/23; 4%) | 4 (4/89; 4%) | 5 (5/112; 4%) | 0 (0/108; 0%) | 0 (0/13; 0%) | **24 (24/260; 9%)** |
| **WCP-Mini13/IgG-ELISA** | 6 (6/6; 100%) | 15 (15/21; 71%) | 1 (1/23; 4%) | 4 (4/89; 4%) | 5 (5/112; 4%) | 0 (0/108; 0%) | 0 (0/13; 0%) | **26 (26/260; 10%)** |
| **TMP-Paidjan/IgG-ELISA** | 6 (6/6; 100%) | 13 (13/21; 62%) | 2 (2/23; 9%) | 8 (8/89; 9%) | 10 (10/112; 9%) | 0 (0/108; 0%) | 0 (0/13; 0%) | **29 (29/260; 11%)** |
| **TMP-Bataviae/IgG-ELISA** | 6 (6/6; 100%) | 12 (12/21; 57%) | 2 (2/23; 9%) | 9 (9/89; 10%) | 11 (11/112; 10%) | 0 (0/108; 0%) | 0 (0/13; 0%) | **29 (29/260; 11%)** |
| **TMP-Mini06/IgG-ELISA** | 6 (6/6; 100%) | 11 (11/21; 52%) | 0 (0/23; 0%) | 0 (0/89; 0%) | 0 (0/112; 0%) | 0 (0/108; 0%) | 0 (0/13; 0%) | **17 (17/260; 7%)** |
| **TMP-Mini13/IgG-ELISA** | 6 (6/6; 100%) | 12 (12/21; 57%) | 0 (0/23; 0%) | 0 (0/89; 0%) | 0 (0/112; 0%) | 0 (0/108; 0%) | 0 (0/13; 0%) | **18 (18/260; 7%)** |
| **OMP-Paidjan/IgG-ELISA** | 6 (6/6; 100%) | 13 (13/21; 62%) | 0 | 0 | | 0 (0/108; 0%) | 0 (0/13; 0%) | **19 (19/260; 7%)** |

| | | | | | | |
|---|---|---|---|---|---|---|
| | | | (0/23; 0%) (0/89; 0%)<br>0 (0/112; 0%) | | | |
| **OMP-Bataviae/IgG-ELISA** | 6 (6/6; 100%) | 9 (9/21; 43%) | 0 (0/23; 0%) 0 (0/89; 0%)<br>0 (0/112; 0%) | 0 (0/108; 0%) | 0 (0/13; 0%) | **15 (15/260; 6%)** |
| **OMP-Mini06/IgG-ELISA** | 6 (6/6; 100%) | 10 (10/21; 48%) | 0 (0/23; 0%) 0 (0/89; 0%)<br>0 (0/112; 0%) | 0 (0/108; 0%) | 0 (0/13; 0%) | **16 (16/260; 6%)** |
| **OMP-Mini13/IgG-ELISA** | 6 (6/6; 100%) | 12 (12/21; 57%) | 0 (0/23; 0%) 0 (0/89; 0%)<br>0 (0/112; 0%) | 0 (0/108; 0%) | 0 (0/13; 0%) | **18 (18/260; 7%)** |
| **Methods (modified ELISAs), protein preparation, and common *Leptospira* serovars in the canine leptospirosis vaccine used in the ELISAs** | | | | | | |
| **WCP-Ictero/IgG-ELISA** | 0 (0/6; 0%) | 0 (0/21; 0%) | 23 (23/23; 100%) 74 (74/89; 83%)<br>97 (97/112; 87%) | 31 (31/108; 29%) | 0 (0/13; 0%) | **128 (128/260; 49%)** |
| **WCP-Pom/IgG-ELISA** | 0 (0/6; 0%) | 0 (0/21; 0%) | 23 (23/23; 100%) 67 (67/89; 75%)<br>90 (90/112; 80%) | 28 (28/108; 26%) | 0 (0/13; 0%) | **118 (118/260; 43%)** |
| **WCP-Grip/IgG-ELISA** | 0 (0/6; 0%) | 0 (0/21; 0%) | 23 (23/23; 100%) 70 (70/89; 79%)<br>93 (93/112; 83%) | 29 (29/108; 27%) | 0 (0/13; 0%) | **122 (122/260; 47%)** |
| **WCP-Can/IgG-ELISA** | 0 (0/6; 0%) | 0 (0/21; 0%) | 23 (23/23; 100%) 77 (77/89; 87%)<br>100 (100/112; 89%) | 34 (34/108; 31%) | 0 (0/13; 0%) | **134 (134/260; 52%)** |
| **TMP-Ictero/IgG-ELISA** | 0 (0/6; 0%) | 0 (0/21; 0%) | 20 (20/23; 87%) 69 (69/89; 78%)<br>89 (89/112; 79%) | 29 (29/108; 27%) | 0 (0/13; 0%) | **118 (118/260; 43%)** |
| **TMP-Pom/IgG-ELISA** | 0 (0/6; 0%) | 0 (0/21; 0%) | 18 (18/23; 78%) 64 (64/89; 72%)<br>82 (82/112; 73%) | 25 (25/108; 23%) | 0 (0/13; 0%) | **107 (107/260; 41%)** |
| **TMP-Grip/IgG-ELISA** | 0 (0/6; 0%) | 0 (0/21; 0%) | 19 (19/23; 83%) 66 (66/89; 74%)<br>85 (85/112; 76%) | 27 (27/108; 25%) | 0 (0/13; 0%) | **112 (112/260; 43%)** |
| **TMP-Can/IgG-ELISA** | 0 (0/6; 0%) | 0 (0/21; 0%) | 20 (20/23; 87%) 72 (72/89; 81%)<br>92 (92/112; 82%) | 31 (31/108; 29%) | 0 (0/13; 0%) | **123 (123/260; 47%)** |
| **OMP-Ictero/IgG-ELISA** | 0 (0/6; 0%) | 0 (0/21; 0%) | 17 (17/23; 74%) 64 (64/89; 72%)<br>81 (81/112; 72%) | 26 (26/108; 24%) | 0 (0/13; 0%) | **107 (107/260; 41%)** |
| **OMP-Pom/IgG-ELISA** | 0 (0/6; 0%) | 0 (0/21; 0%) | 15 (15/23; 65%) 59 (74/89; 66%)<br>74 (74/112; 66%) | 23 (23/108; 21%) | 0 (0/13; 0%) | **97 (97/260; 37%)** |
| **OMP-Grip/IgG-ELISA** | 0 (0/6; 0%) | 0 (0/21; 0%) | 16 (16/23; 70%) 61 (61/89; 69%) | 24 (24/108; 22%) | 0 (0/13; 0%) | **101 (101/260; 39%)** |

| | | | | | | | |
|---|---|---|---|---|---|---|---|
| | | | 77 (77/112; 69%) | | | | |
| **OMP-Can/IgG-ELISA** | 0 (0/6; 0%) | 0 (0/21; 0%) | 17 (17/23; 74%) | 68 (68/89; 76%) | 29 (29/108; 27%) | 0 (0/13; 0%) | **114 (114/260; 44%)** |
| | | | 85 (85/112; 76%) | | | | |

N/A; Not applicable. WCP; whole-cell protein, TMP; total membrane protein, OMP; outer membrane protein. Ictero; Icterohaemorrhagiae, Pom; Pomona, Grip; Grippotyphosa, Can; Canicola. *; Twenty-three serum samples from group 3 were examined by both MAT and ELISAs and used for preliminary evaluation of the effectiveness of diagnostic performance of the modified ELISAs. **; Another 89 serum samples were examined only by ELISAs and were used to screen for leptospiral IgG antibody and confirm the usefulness of the modified ELISAs.

**Supplementary Table S2.** Evaluation of diagnostic tests, including sensitivity, specificity, PPV, NPV, accuracy and repeatability, and reproducibility (intra- & inter-assay), among the modified ELISAs using the five local Thai isolates of *Leptospira* serovars with 50 sera from three groups of dogs (groups 1, 2 and 3) compared to serum PCR and MAT results.

| IgG ELISAs | Evaluation of diagnostic tests | | | | | | | | | | | | | | |
|---|---|---|---|---|---|---|---|---|---|---|---|---|---|---|---|
| | Comparison with PCR from urine (n = 27) | | | | | Comparison with PCR from sera (n = 50) | | | | | Comparison with MAT from sera (n = 50) | | | | |
| | Sensitivity (%) | Specificity (%) | PPV (%) | NPV (%) | Accuracy (%) | Sensitivity (%) | Specificity (%) | PPV (%) | NPV (%) | Accuracy (%) | Sensitivity (%) | Specificity (%) | PPV (%) | NPV (%) | Accuracy (%) |
| **WCP-Paidjan** | 100 | 38.1 | 31.6 | 100 | 51.9 | 100 | 59.1 | 25.0 | 100 | 64.0 | 57.1 | 55.6 | 33.3 | 76.9 | 56.0 |
| **WCP-Dadas** | 100 | 33.3 | 30 | 100 | 48.1 | 100 | 40.9 | 18.8 | 100 | 48.0 | 85.7 | 44.4 | 37.5 | 88.9 | 56.0 |
| **WCP-Bataviae** | 100 | 33.3 | 30 | 100 | 48.1 | 100 | 65.9 | 28.6 | 100 | 70.0 | 57.1 | 63.9 | 38.1 | 79.3 | 62.0 |
| **WCP-Mini06** | 100 | 38.1 | 31.6 | 100 | 51.8 | 100 | 68.2 | 30.0 | 100 | 72.0 | 57.1 | 66.7 | 40.0 | 80.0 | 64.0 |
| **WCP-Mini13** | 100 | 28.6 | 28.6 | 100 | 44.4 | 100 | 63.6 | 27.3 | 100 | 68.0 | 64.3 | 63.9 | 40.9 | 82.1 | 64.0 |
| **TMP-Paidjan** | 100 | 38.1 | 31.6 | 100 | 51.9 | 100 | 65.9 | 28.6 | 100 | 70.0 | 64.3 | 66.7 | 42.9 | 82.8 | 58.0 |
| **TMP-Dadas** | 100 | 38.1 | 31.6 | 100 | 51.9 | 100 | 56.8 | 24.0 | 100 | 62.0 | 64.3 | 55.6 | 36.0 | 80.0 | 66.0 |
| **TMP-Bataviae** | 100 | 42.9 | 33.3 | 100 | 55.6 | 100 | 68.9 | 30.0 | 100 | 72.0 | 57.1 | 66.7 | 40.0 | 80.0 | 64.0 |
| **TMP-Mini06** | 100 | 47.6 | 35.3 | 100 | 59.3 | 100 | 75.0 | 35.3 | 100 | 78.0 | 42.9 | 69.4 | 35.3 | 75.8 | 62.0 |
| **TMP-Mini13** | 100 | 42.9 | 33.3 | 100 | 55.6 | 100 | 72.7 | 33.3 | 100 | 76.0 | 50.0 | 69.4 | 38.9 | 78.1 | 64.0 |
| **OMP-Paidjan** | 100 | 38.1 | 31.6 | 100 | 51.9 | 100 | 70.5 | 31.6 | 100 | 74.0 | 57.1 | 69.4 | 42.1 | 80.7 | 66.0 |
| **OMP-Dadas** | 100 | 28.6 | 28.6 | 100 | 44.4 | 100 | 65.9 | 28.6 | 100 | 70.0 | 64.3 | 66.7 | 42.9 | 82.8 | 66.0 |
| **OMP-Bataviae** | 100 | 57.1 | 40 | 100 | 54.5 | 100 | 79.6 | 40.0 | 100 | 82.0 | 35.7 | 72.2 | 33.3 | 74.3 | 62.0 |
| **OMP-Mini06** | 100 | 52.4 | 37.5 | 100 | 63.0 | 100 | 77.3 | 37.5 | 100 | 80.0 | 42.9 | 72.2 | 37.5 | 76.5 | 64.0 |
| **OMP-Mini13** | 100 | 42.9 | 33.3 | 100 | 55.6 | 100 | 72.7 | 33.3 | 100 | 76.0 | 50.0 | 69.4 | 38.9 | 78.1 | 64.0 |

| IgG-ELISA assays | Diagnostic precision | |
|---|---|---|
| | Intra-assay/repeatability (%) | Inter-assay/reproducibility (%) |
| **WCP/IgG-ELISAs** | 6.3 | 11.0 |
| **TMP/IgG-ELISAs** | 4.1 | 9.3 |
| **OMP/IgG-ELISAs** | 2.6 | 8.5 |

**Supplementary Table S3.** Cohen's kappa statistics of modified ELISAs against PCR (agent detection) and MAT (antibody detection) for 50 sera from three dog groups (groups 1, 2, and 3).

| ELISAs | Cohen's kappa index | | |
|---|---|---|---|
| | **Comparison with PCR from urine (n=27)** | **Comparison with PCR from sera (n=50)** | **Comparison with MAT from sera (n=50)** |
| WCP-Paidjan/IgG-ELISA | 0.215 | 0.257 | 0.104 |
| WCP-Dadas/IgG-ELISA | 0.182 | 0.142 | 0.217 |
| WCP-Bataviae/IgG-ELISA | 0.182 | 0.317 | 0.182 |
| WCP-Mini06/IgG-ELISA | 0.215 | 0.340 | 0.211 |
| WCP-Mini13/IgG-ELISA | 0.151 | 0.300 | 0.240 |
| TMP-Paidjan/IgG-ELISA | 0.215 | 0.317 | 0.269 |
| TMP-Dadas/IgG-ELISA | 0.215 | 0.240 | 0.160 |
| TMP-Bataviae/IgG-ELISA | 0.250 | 0.340 | 0.211 |
| TMP-Mini06/IgG-ELISA | 0.288 | 0.419 | 0.115 |
| TMP-Mini13/IgG-ELISA | 0.250 | 0.390 | 0.179 |
| OMP-Paidjan/IgG-ELISA | 0.215 | 0.364 | 0.240 |
| OMP-Dadas/IgG-ELISA | 0.151 | 0.317 | 0.269 |
| OMP-Bataviae/IgG-ELISA | 0.372 | 0.483 | 0.078 |
| OMP-Mini06/IgG-ELISA | 0.328 | 0.449 | 0.144 |
| OMP-Mini13/IgG-ELISA | 0.250 | 0.390 | 0.179 |

A)

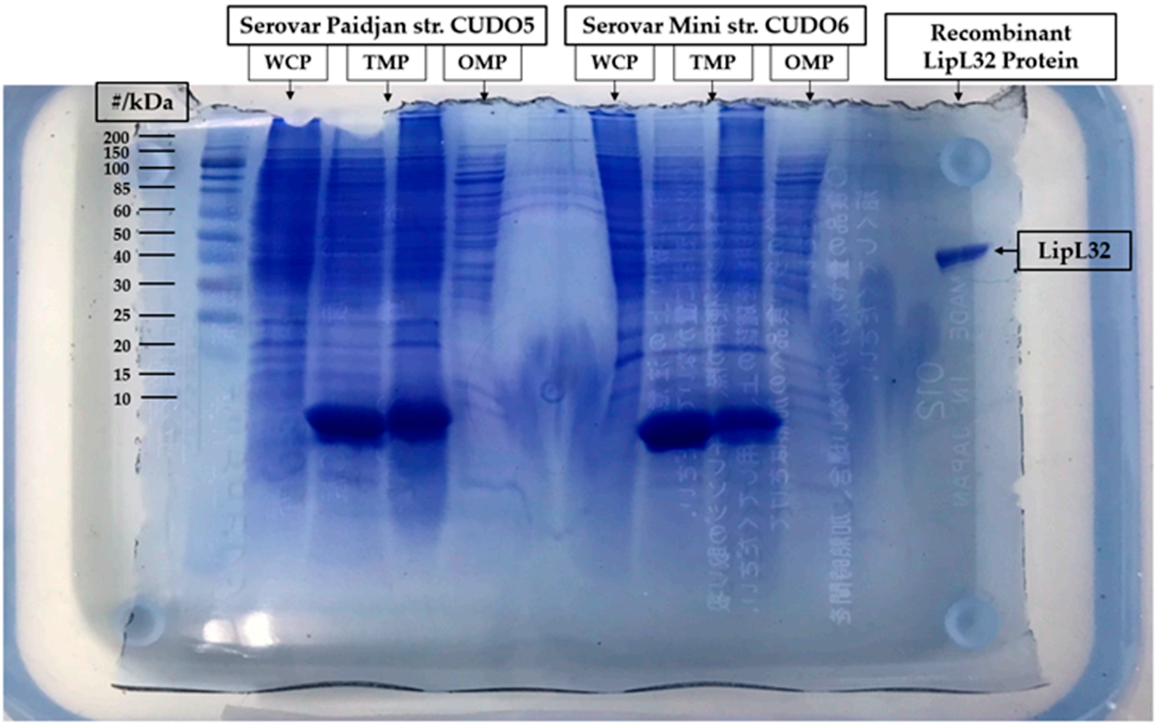
Serovar Paidjan str. CUDO5
WCP
TMP
OMP
Serovar Mini str. CUDO6
WCP
TMP
OMP
Recombinant
LipL32 Protein
#/kDa
200
150
100
85
60
50
40
30
25
20
15
10
LipL32

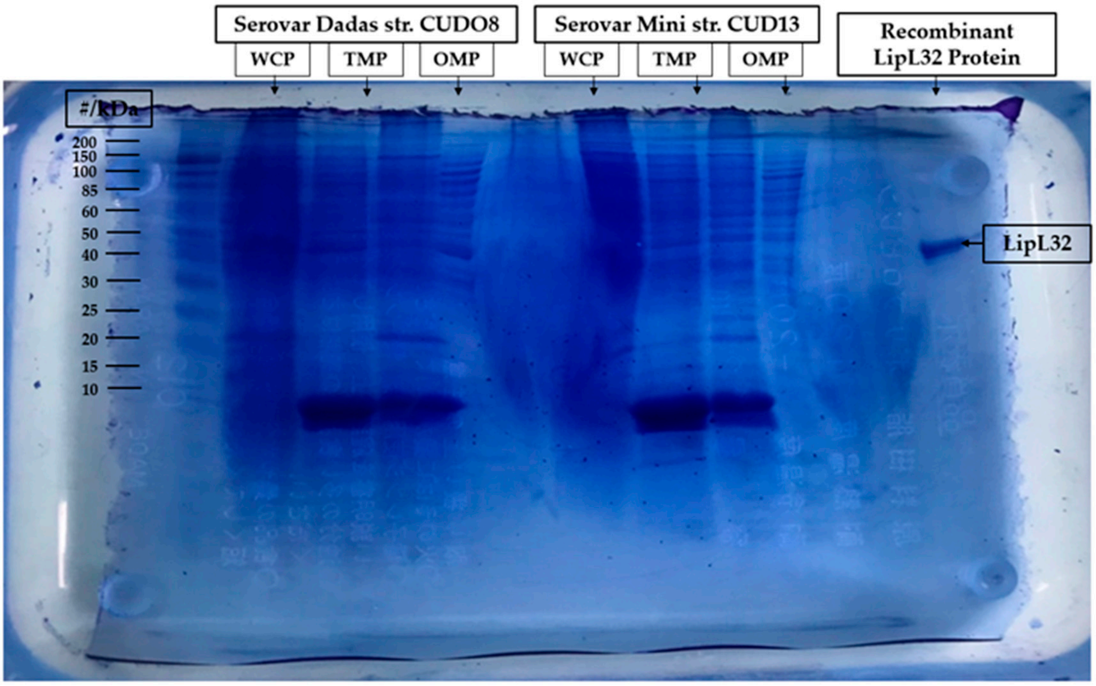
Serovar Dadas str. CUDO8
WCP
TMP
OMP
Serovar Mini str. CUD13
WCP
TMP
OMP
Recombinant
LipL32 Protein
#/kDa
200
150
100
85
60
50
40
30
25
20
15
10
LipL32

B)

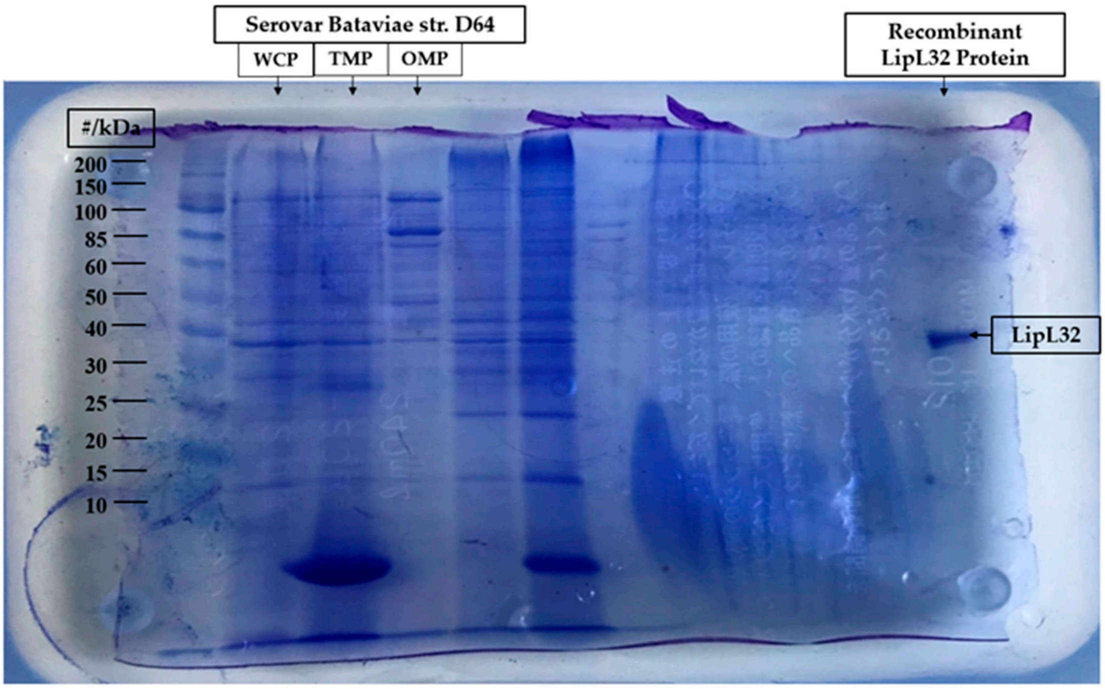

Serovar Bataviae str. D64
WCP
TMP
OMP
Recombinant LipL32 Protein
#/kDa
200
150
100
85
60
50
40
30
25
20
15
10
LipL32


C)

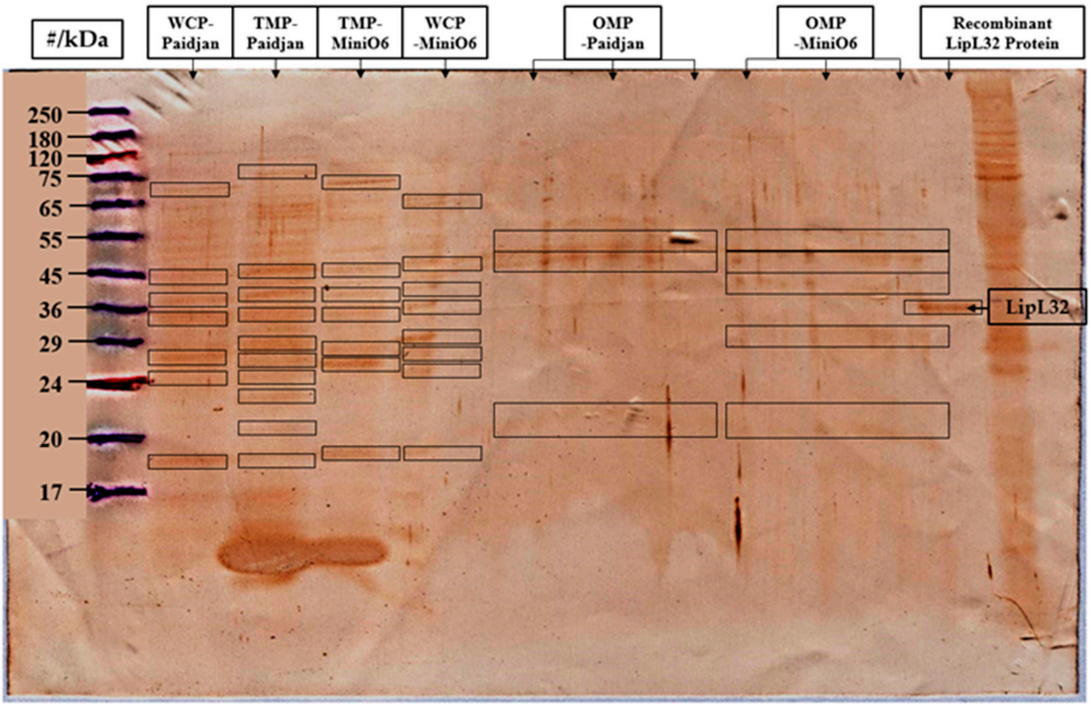

#/kDa
WCP-Paidjan
TMP-Paidjan
TMP-MiniO6
WCP -MiniO6
OMP -Paidjan
OMP -MiniO6
Recombinant LipL32 Protein
250
180
120
75
65
55
45
36
29
24
20
17
LipL32


D)

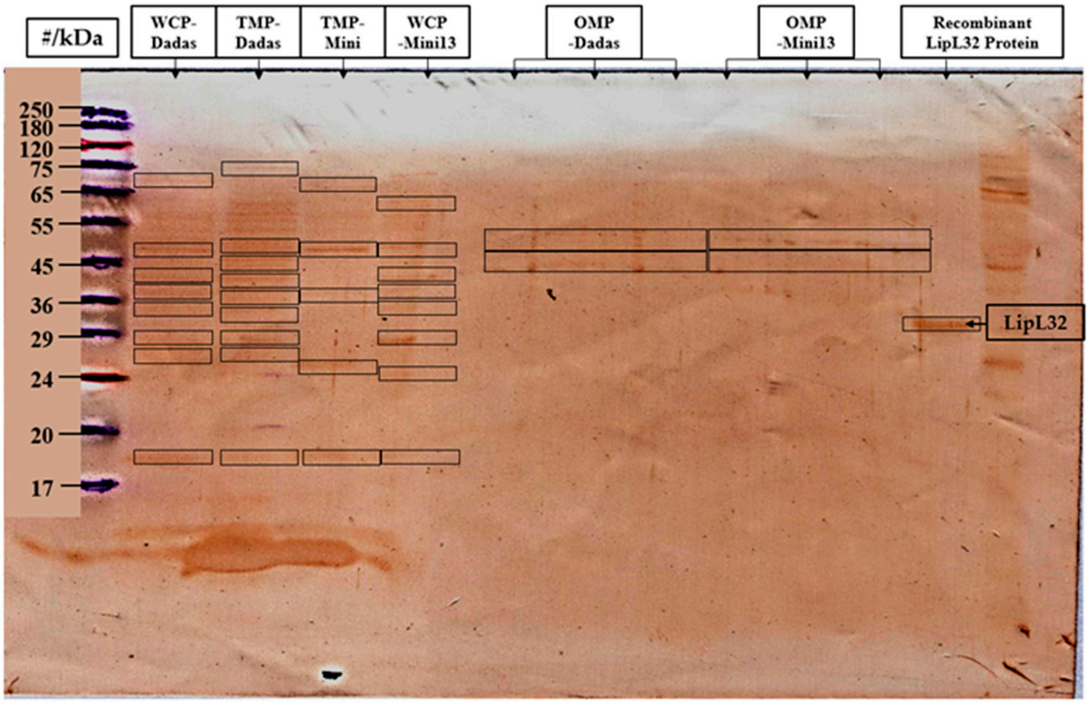

E)

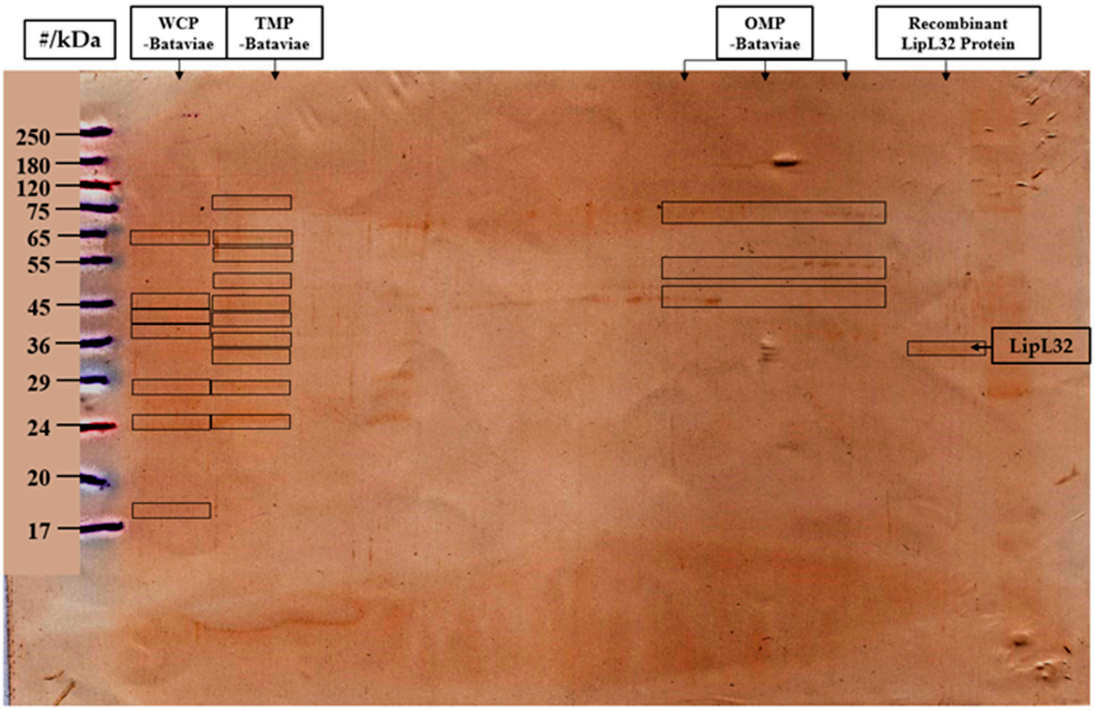

F)

**Supplementary Figure S1.** Protein component confirmation of the five local Thai isolates of *Leptospira interrogans* and *Leptospira weilii* serovars, including serovars Paidjan, Dadas, Bataviae, Mini06, and Mini13 by 12.5% SDS-PAGE with Coomassie Brilliant Blue R-250 (A, B, and C) and Western blotting with serum from a dog confirmed as positive for leptospirosis by isolation and PCR, which was followed by horseradish peroxidase (HRP) conjugated goat anti-dog IgG antibody and visualized by adding DAB substrate (D, E and F).

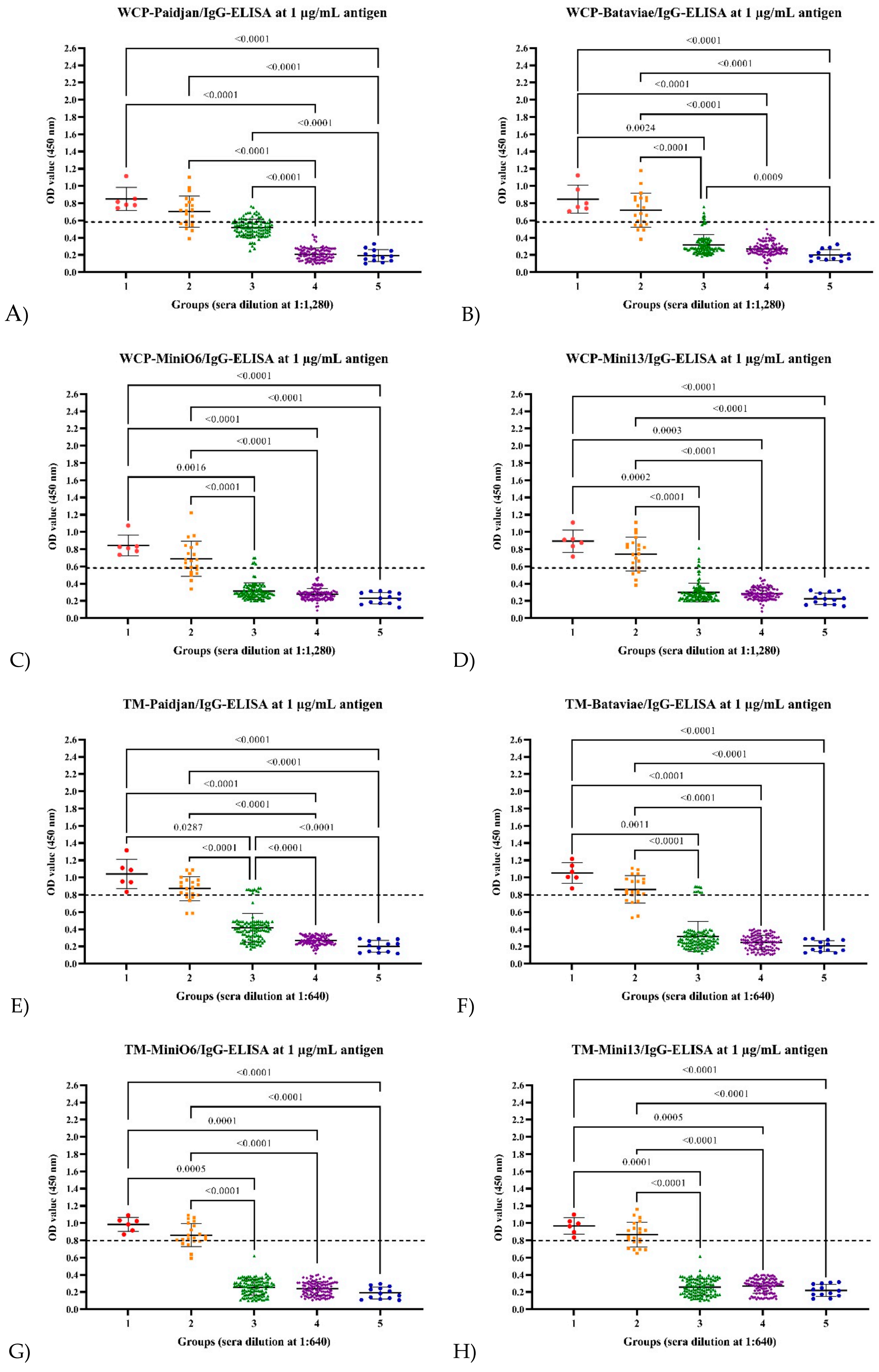

WCP-Paidjan/IgG-ELISA at 1 µg/mL antigen
<0.0001
<0.0001
<0.0001
<0.0001
<0.0001
<0.0001
OD value (450 nm)
Groups (sera dilution at 1:1,280)
A)
WCP-Bataviae/IgG-ELISA at 1 µg/mL antigen
<0.0001
<0.0001
<0.0001
<0.0001
0.0024
<0.0001
0.0009
OD value (450 nm)
Groups (sera dilution at 1:1,280)
B)
WCP-Mini06/IgG-ELISA at 1 µg/mL antigen
<0.0001
<0.0001
<0.0001
<0.0001
0.0016
<0.0001
OD value (450 nm)
Groups (sera dilution at 1:1,280)
C)
WCP-Mini13/IgG-ELISA at 1 µg/mL antigen
<0.0001
<0.0001
0.0003
<0.0001
0.0002
<0.0001
OD value (450 nm)
Groups (sera dilution at 1:1,280)
D)
TM-Paidjan/IgG-ELISA at 1 µg/mL antigen
<0.0001
<0.0001
<0.0001
<0.0001
0.0287
<0.0001
<0.0001
<0.0001
OD value (450 nm)
Groups (sera dilution at 1:640)
E)
TM-Bataviae/IgG-ELISA at 1 µg/mL antigen
<0.0001
<0.0001
<0.0001
<0.0001
0.0011
<0.0001
OD value (450 nm)
Groups (sera dilution at 1:640)
F)
TM-Mini06/IgG-ELISA at 1 µg/mL antigen
<0.0001
<0.0001
0.0001
<0.0001
0.0005
<0.0001
OD value (450 nm)
Groups (sera dilution at 1:640)
G)
TM-Mini13/IgG-ELISA at 1 µg/mL antigen
<0.0001
<0.0001
0.0005
<0.0001
0.0001
<0.0001
OD value (450 nm)
Groups (sera dilution at 1:640)
H)

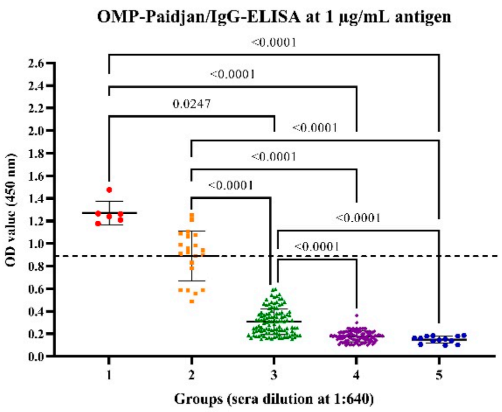


I)

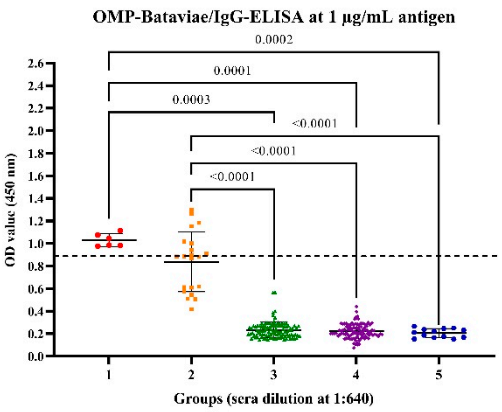


J)

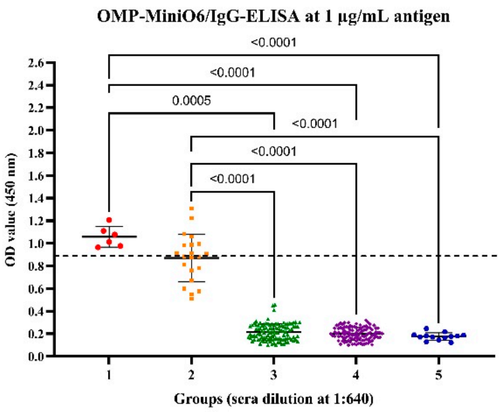


K)

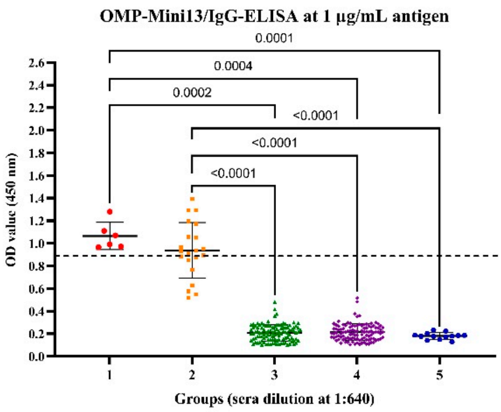


L)

**Supplementary Figure S2.** The levels of IgG antibody detected in modified ELISAs against whole-cell protein (WCP), total membrane protein (TMP), and outer membrane protein (OMP) from the other four local Thai isolates of *Leptospira* serovars, including Paidjan, Bataviae, Mini06, and Mini13, at 1:1,280, 1:640 and 1:640 sera dilutions. Comparisons among 260 sera from five groups consisting of dogs from Nan province confirmed as infected by PCR and isolation (Group 1), unvaccinated dogs from Nan province (Group 2), vaccinated dogs from Bangkok (Group 3), unvaccinated dogs from non-endemic areas (Group 4), and unvaccinated puppies from non-endemic areas (Group 5).

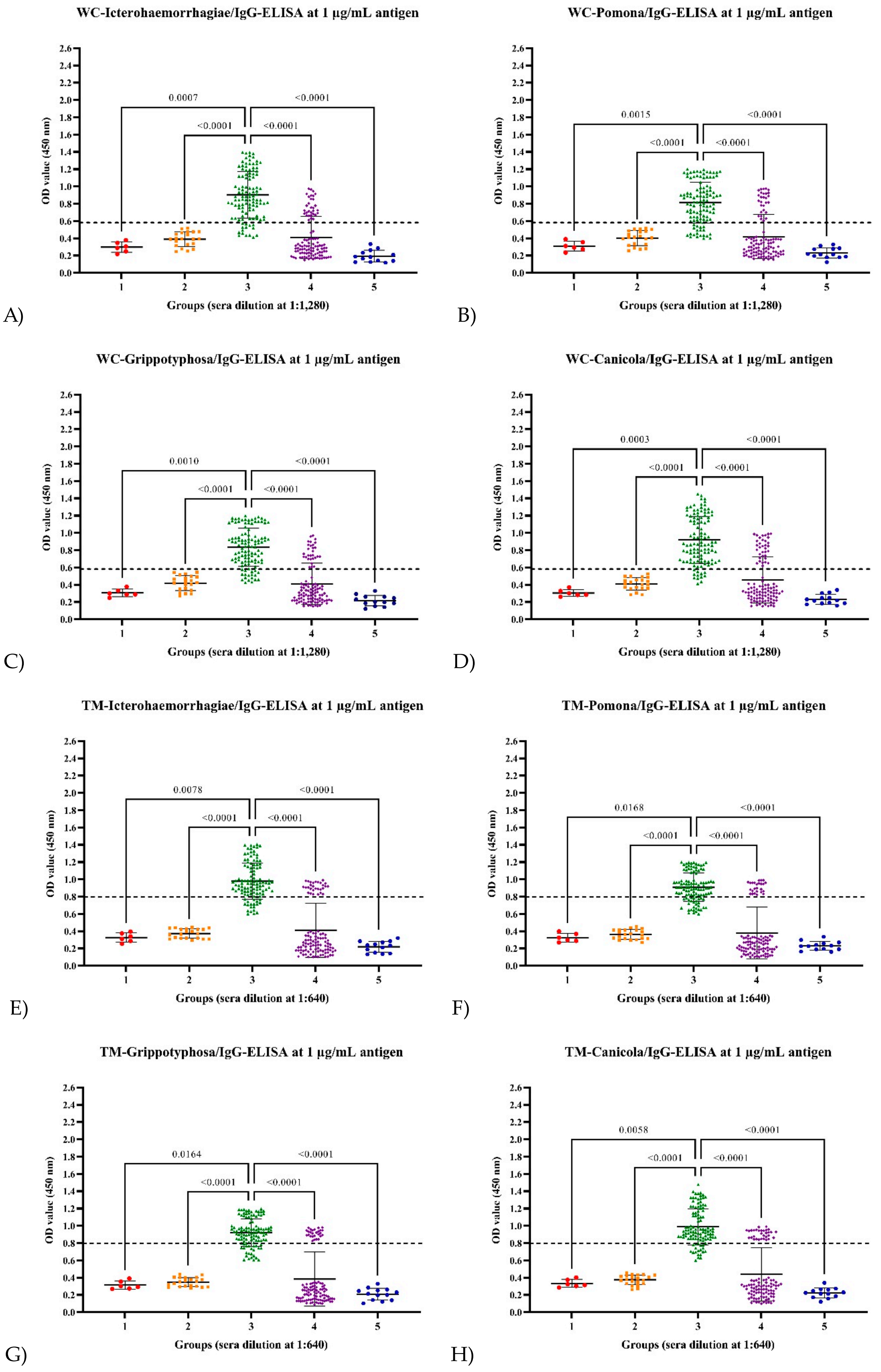
WC-Icterohaemorrhagiae/IgG-ELISA at 1 µg/mL antigen
OD value (450 nm)
0.0007
<0.0001
<0.0001
<0.0001
Groups (sera dilution at 1:1,280)
A)
WC-Pomona/IgG-ELISA at 1 µg/mL antigen
OD value (450 nm)
0.0015
<0.0001
<0.0001
<0.0001
Groups (sera dilution at 1:1,280)
B)
WC-Grippotyphosa/IgG-ELISA at 1 µg/mL antigen
OD value (450 nm)
0.0010
<0.0001
<0.0001
<0.0001
Groups (sera dilution at 1:1,280)
C)
WC-Canicola/IgG-ELISA at 1 µg/mL antigen
OD value (450 nm)
0.0003
<0.0001
<0.0001
<0.0001
Groups (sera dilution at 1:1,280)
D)
TM-Icterohaemorrhagiae/IgG-ELISA at 1 µg/mL antigen
OD value (450 nm)
0.0078
<0.0001
<0.0001
<0.0001
Groups (sera dilution at 1:640)
E)
TM-Pomona/IgG-ELISA at 1 µg/mL antigen
OD value (450 nm)
0.0168
<0.0001
<0.0001
<0.0001
Groups (sera dilution at 1:640)
F)
TM-Grippotyphosa/IgG-ELISA at 1 µg/mL antigen
OD value (450 nm)
0.0164
<0.0001
<0.0001
<0.0001
Groups (sera dilution at 1:640)
G)
TM-Canicola/IgG-ELISA at 1 µg/mL antigen
OD value (450 nm)
0.0058
<0.0001
<0.0001
<0.0001
Groups (sera dilution at 1:640)
H)

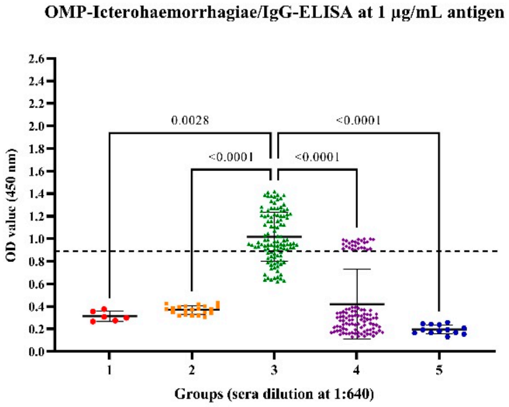


I)

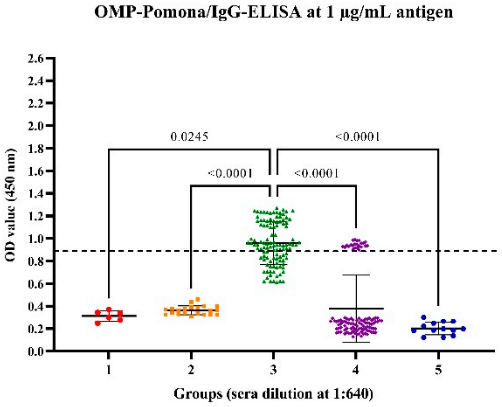


J)

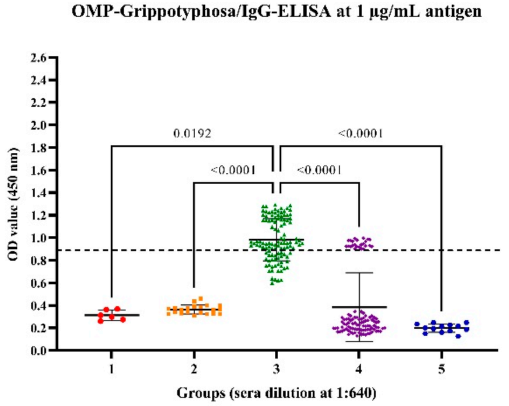


K)

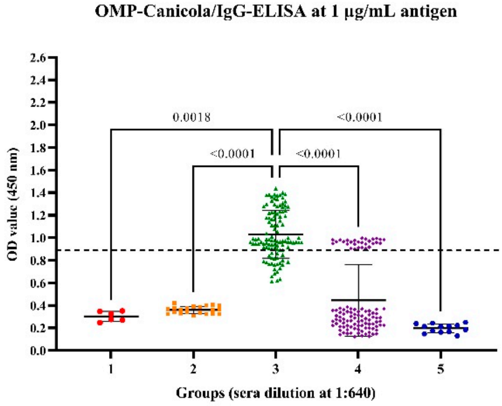


L)

**Supplementary Figure S3.** The levels of IgG antibody in modified ELISAs against whole-cell protein (WCP), total membrane proteins (TMP), and outer membrane protein (OMP) preparations from four common *Leptospira* serovars used in the leptospirosis vaccine for dogs, including Icterohaemorrhagiae, Pomona, Grippotyphosa and Canicola at 1:1,280, 1:640 and 1:640 sera dilutions. Comparisons among 260 sera from five groups consisting of dogs from Nan province confirmed as infected by PCR and isolation (Group 1), unvaccinated dogs from Nan province (Group 2), vaccinated dogs from Bangkok (Group 3), unvaccinated dogs from the non-endemic areas (Group 4), and unvaccinated puppies from non-endemic areas (Group 5).

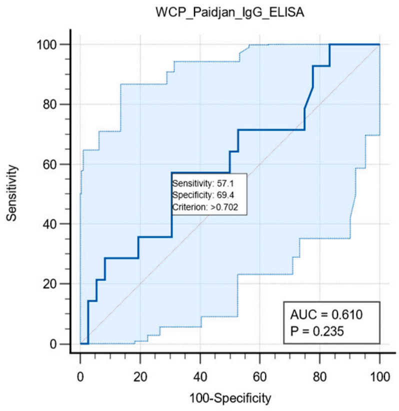
WCP_Paidjan_IgG_ELISA
Sensitivity: 57.1
Specificity: 69.4
Criterion: >0.702
AUC = 0.610
P = 0.235
Sensitivity
100-Specificity

A)

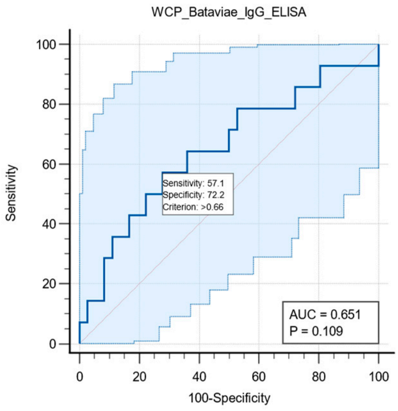
WCP_Bataviae_IgG_ELISA
Sensitivity: 57.1
Specificity: 72.2
Criterion: >0.66
AUC = 0.651
P = 0.109
Sensitivity
100-Specificity

B)

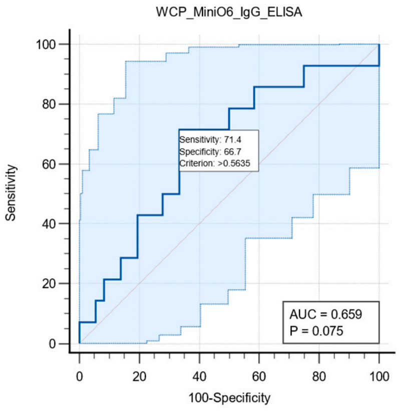
WCP_MiniO6_IgG_ELISA
Sensitivity: 71.4
Specificity: 66.7
Criterion: >0.5635
AUC = 0.659
P = 0.075
Sensitivity
100-Specificity

C)

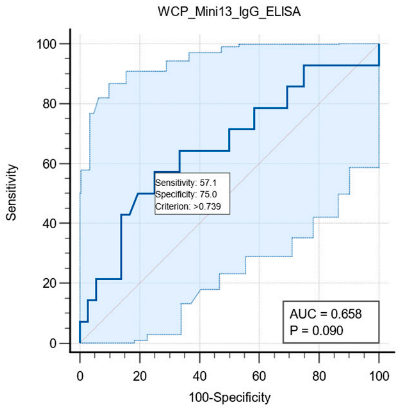
WCP_Mini13_IgG_ELISA
Sensitivity: 57.1
Specificity: 75.0
Criterion: >0.739
AUC = 0.658
P = 0.090
Sensitivity
100-Specificity

D)

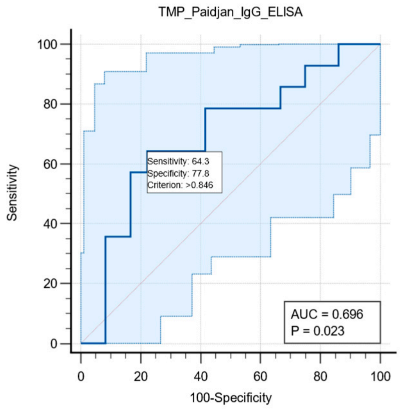
TMP_Paidjan_IgG_ELISA
Sensitivity: 64.3
Specificity: 77.8
Criterion: >0.846
AUC = 0.696
P = 0.023
Sensitivity
100-Specificity

E)

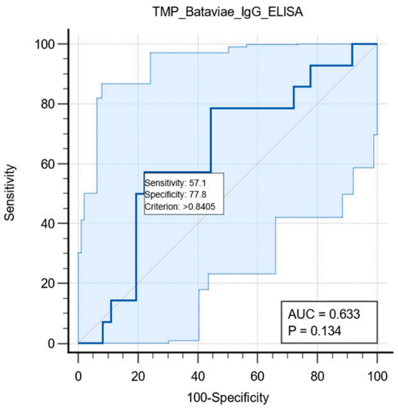
TMP_Bataviae_IgG_ELISA
Sensitivity: 57.1
Specificity: 77.8
Criterion: >0.8405
AUC = 0.633
P = 0.134
Sensitivity
100-Specificity

F)

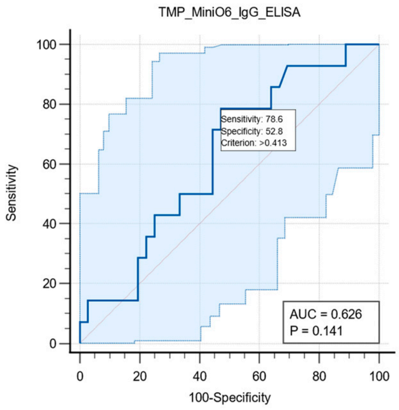
TMP_MiniO6_IgG_ELISA
Sensitivity: 78.6
Specificity: 52.8
Criterion: >0.413
AUC = 0.626
P = 0.141
Sensitivity
100-Specificity

G)

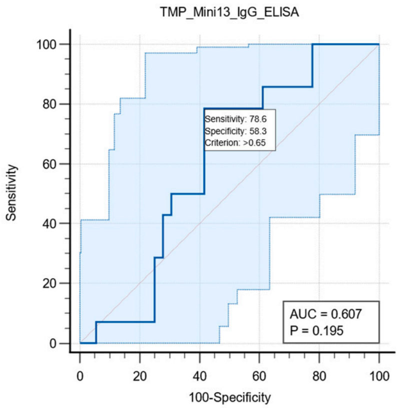
TMP_Mini13_IgG_ELISA
Sensitivity: 78.6
Specificity: 58.3
Criterion: >0.65
AUC = 0.607
P = 0.195
Sensitivity
100-Specificity

H)

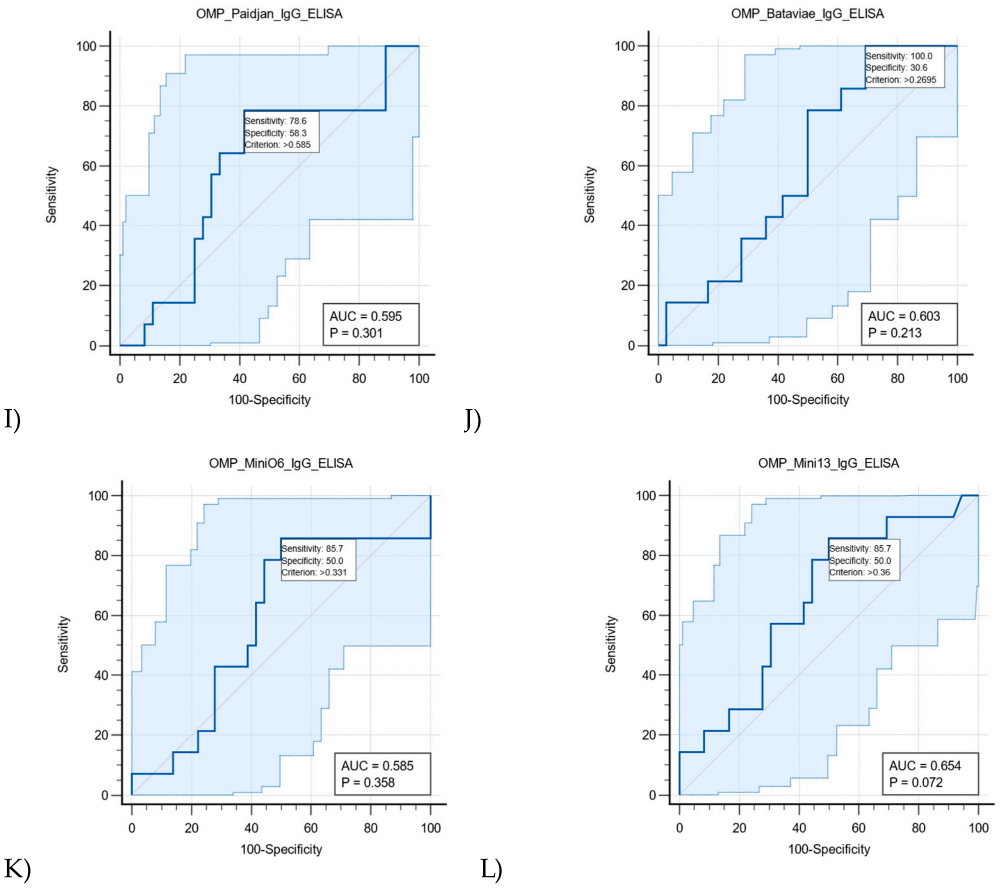


**Supplementary Figure S4.** Receiver–operator curve (ROC) and area under the curve of ROC (AUC) of modified ELISAs against whole-cell protein (WCP), total membrane protein (TMP), and outer membrane protein (OMP) preparations from the four local Thai isolates of *Leptospira* serovars, including Paidjan, Bataviae, Mini06, and Mini13, at 1:1,280, 1:640 and 1:640 sera dilutions, with 50 sera from three groups consisting of dogs from Nan province confirmed as infected by PCR and isolation (Group 1), unvaccinated dogs from Nan province (Group 2), and vaccinated dogs from Bangkok (Group 3).

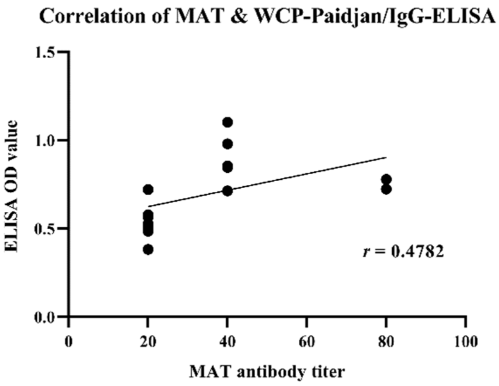

Correlation of MAT & WCP-Paidjan/IgG-ELISA
ELISA OD value
MAT antibody titer
r = 0.4782


A)

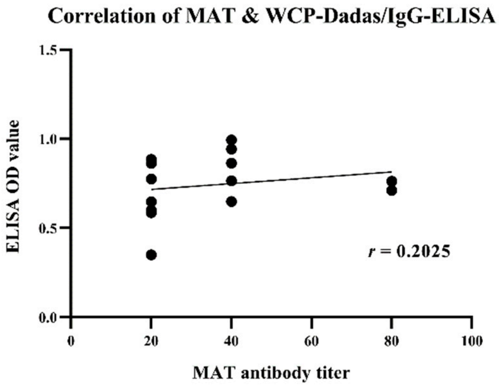

Correlation of MAT & WCP-Dadas/IgG-ELISA
ELISA OD value
MAT antibody titer
r = 0.2025


B)

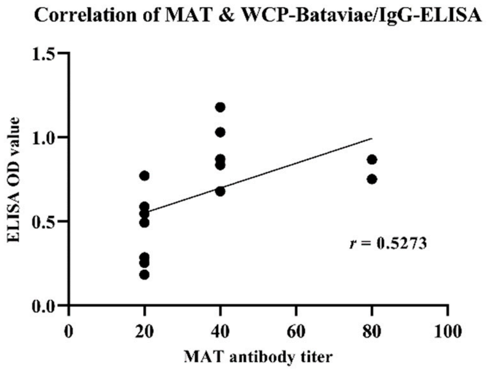

Correlation of MAT & WCP-Bataviae/IgG-ELISA
ELISA OD value
MAT antibody titer
r = 0.5273


C)

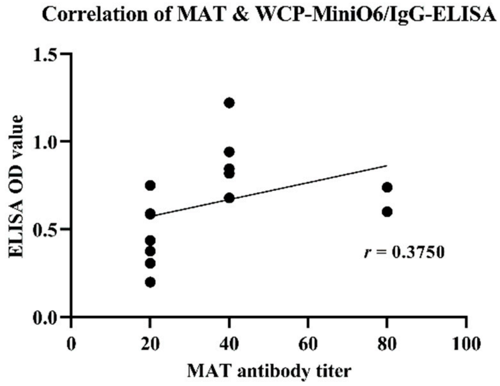

Correlation of MAT & WCP-MiniO6/IgG-ELISA
ELISA OD value
MAT antibody titer
r = 0.3750


D)

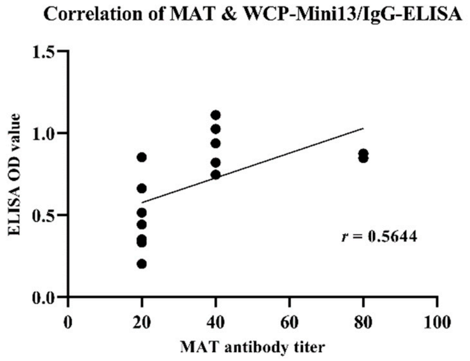

Correlation of MAT & WCP-Mini13/IgG-ELISA
ELISA OD value
MAT antibody titer
r = 0.5644


E)

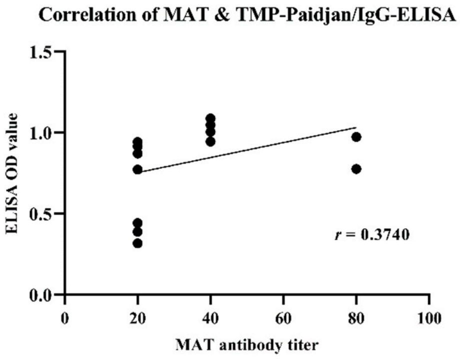

Correlation of MAT & TMP-Paidjan/IgG-ELISA
ELISA OD value
MAT antibody titer
r = 0.3740


F)

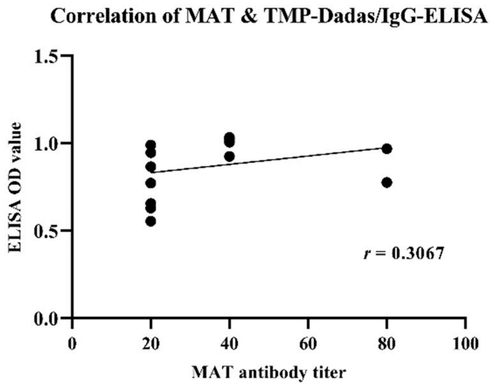

Correlation of MAT & TMP-Dadas/IgG-ELISA
ELISA OD value
MAT antibody titer
r = 0.3067


G)

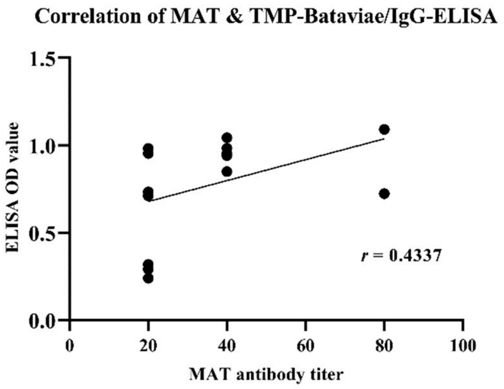

Correlation of MAT & TMP-Bataviae/IgG-ELISA
ELISA OD value
MAT antibody titer
r = 0.4337


H)

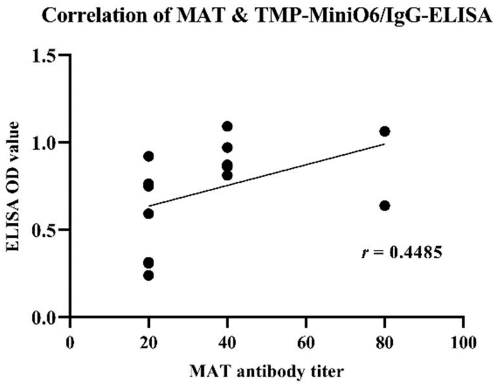


I)

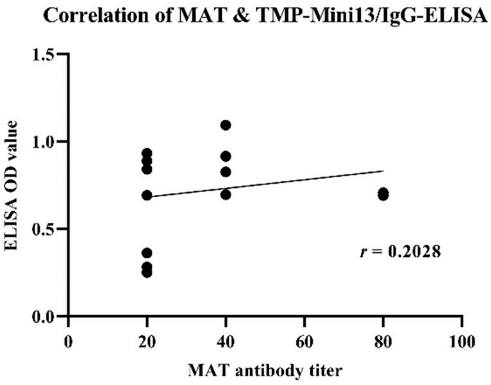


J)

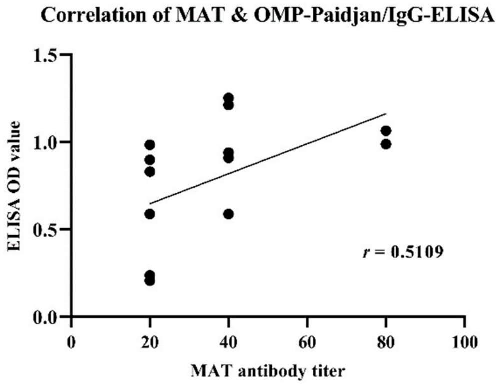


K)

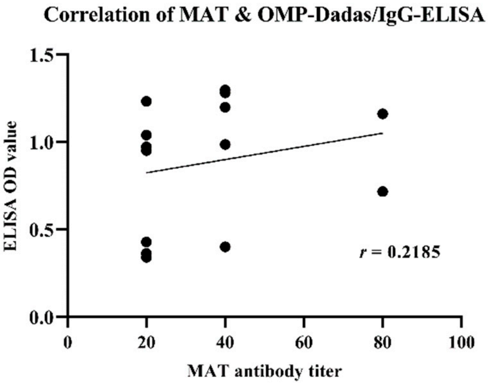


L)

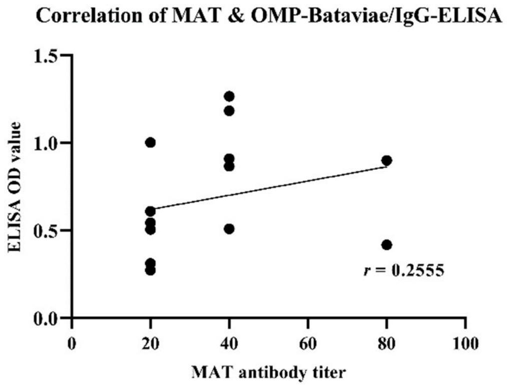


M)

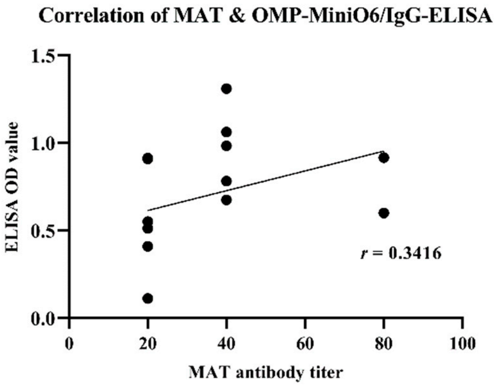


N)

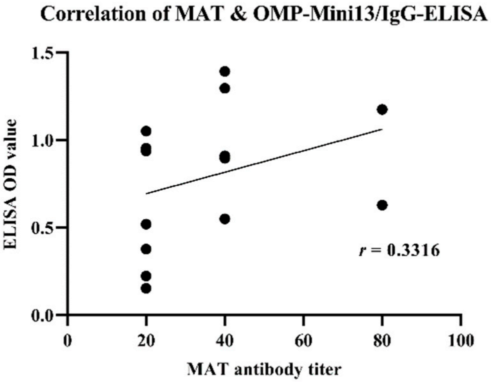


O)

**Supplementary Figure S5.** Pearson correlation of antibody titer in MAT and antibody level in modified ELISAs using 14 sera with MAT titers above 1:20 dilution obtained from dogs in groups 1, 2, and 3.
